# Risk of subsequent lower respiratory tract infection (LRTI) after hospitalization for COVID-19 LRTI and non-COVID-19 LRTI: a retrospective cohort study

**DOI:** 10.1186/s41479-023-00117-5

**Published:** 2023-10-05

**Authors:** Katia J. Bruxvoort, Heidi Fischer, Joseph A. Lewnard, Vennis X. Hong, Magdalena Pomichowski, Lindsay R. Grant, Luis Jódar, Bradford D. Gessner, Sara Y. Tartof

**Affiliations:** 1 Department of Research & Evaluation, Kaiser Permanente Southern California, Pasadena, CA USA; 2 Department of Epidemiology, School of Public Health, University of Alabama at Birmingham, 1665 University Blvd, Birmingham, AL 35233 USA; 3 Division of Epidemiology, School of Public Health, University of California, Berkeley, Berkeley, CA USA; 4 Division of Infectious Diseases & Vaccinology, School of Public Health, University of California, Berkeley, Berkeley, CA USA; 5 Center for Computational Biology, College of Engineering, University of California, Berkeley, Berkeley, CA USA; 6 Pfizer Vaccines, Collegeville, PA USA

**Keywords:** Pneumonia, Lower respiratory tract infection, COVID-19, Hospitalization

## Abstract

**Background:**

Respiratory pathogens, including SARS-CoV-2, can cause pulmonary structural damage and physiologic impairment, which may increase the risk of subsequent lower respiratory tract infections (LRTI). Prior hospitalization for any reason is a risk factor for LRTI, but data on the risk of subsequent new-onset LRTI following hospitalization for COVID-19 LRTI or non-COVID-19 LRTI are needed to inform strategies for immunizations targeting respiratory pathogens.

**Methods:**

We conducted a retrospective cohort study at Kaiser Permanente Southern California (KPSC) among adults hospitalized from 3/1/2020 to 5/31/2022, excluding labor and delivery. We categorized individuals into 3 mutually exclusive baseline exposure groups: those hospitalized for COVID-19 LRTI, those hospitalized for non-COVID-19 LRTI, and those hospitalized for all other causes without LRTI or COVID-19 (“non-LRTI”). Following hospital discharge, patients were followed up for new-onset LRTI, beginning 30 antibiotic-free days after hospital discharge until 8/31/2022. We used multivariable cause-specific Cox regression with time-varying covariates to estimate hazard ratios (HR) of new-onset LRTI comparing those hospitalized for COVID-19 LRTI or non-COVID-19 LRTI to those hospitalized for non-LRTI, adjusting for demographic and clinical characteristics.

**Results:**

The study included 22,417 individuals hospitalized for COVID-19 LRTI, 12,795 individuals hospitalized for non-COVID-19 LRTI, and 176,788 individuals hospitalized for non-LRTI. Individuals hospitalized for non-COVID-19 LRTI were older and had more comorbidities than those hospitalized for COVID-19 LRTI or non-LRTI. Incidence rates per 1,000 person-years (95% CI) of new-onset LRTI were 52.5 (51.4–53.6) among individuals hospitalized for COVID-19 LRTI, 253.5 (243.7–263.6) among those hospitalized for non-COVID-19 LRTI, and 52.5 (51.4–53.6) among those hospitalized for non-LRTI. The adjusted hazard of new-onset LRTI during follow-up was 20% higher among individuals hospitalized for COVID-19 LRTI (HR 1.20 [95% CI: 1.12–1.28]) and 301% higher among individuals hospitalized for non-COVID-19 LRTI (HR 3.01 [95% CI: 2.87–3.15]) compared to those hospitalized for non-LRTI.

**Conclusion:**

The risk of new-onset LRTI following hospital discharge was high, particularly among those hospitalized for non-COVID-19 LRTI, but also for COVID-19 LRTI. These data suggest that immunizations targeting respiratory pathogens, including COVID-19, should be considered for adults hospitalized for LRTI prior to hospital discharge.

**Supplementary Information:**

The online version contains supplementary material available at 10.1186/s41479-023-00117-5.

## Introduction

Pneumonia is a common cause of morbidity and mortality among adults, and age-associated immunosenescence and the presence of comorbidities dramatically increase pneumonia risk [1]. Specifically, the incidence of hospitalization for community-acquired pneumonia among individuals aged ≥ 85 years in the United States (US) is almost 60-fold greater than among adults aged 18 to 24 years [2]. Adults who have one or more comorbidities have a marked increase in the rate of pneumococcal disease compared with healthy adults [3], and the prevalence of these conditions increases with age [4]. The US Centers for Disease Control and Prevention (CDC) has defined risk factors for pneumococcal disease that comprise chronic heart, lung, and liver disease, diabetes mellitus, smoking, alcoholism, and immunocompromising conditions [5]. In turn, the US Advisory Committee on Immunization Practices (ACIP) has recommended pneumococcal vaccines for these populations [6, 7]. In other countries, public health agencies have identified similar risk factors and recommending bodies have also defined vaccination policy targeting these groups [1].

One risk factor for pneumonia that is less recognized in national guidance is prior infection with influenza or pneumococcus, including hospitalization for previous pneumonia or lower respiratory tract infection (LRTI) [8]. As early as 1982, a study identified previous hospitalization for any reason as a risk factor for readmission or death caused by a subsequent pneumonia [9]. However, data on the risk of pneumonia following a previous LRTI hospitalization are scarce. Similarly, data are limited on the risk of subsequent pneumonia following a hospitalization for COVID-19 LRTI, although there is a growing body of evidence that SARS-CoV-2 causes substantial and persistent physiological and structural lung damage [10–19], which may increase susceptibility to subsequent viral or bacterial infections.

The degree of LRTI risk after hospitalization has important policy implications for immunizations targeting respiratory pathogens. Currently, older US adults and those with comorbidities are recommended to receive 20-valent pneumococcal conjugate vaccine (PCV20) or 15-valent pneumococcal conjugate vaccine (PCV15) plus 23-valent pneumococcal polysaccharide vaccine (PPSV23) as well as seasonal influenza vaccine and COVID-19 vaccine [20, 21]. Other respiratory vaccines may soon be available including those for preventing respiratory syncytial virus, parainfluenza, and human metapneumovirus. If hospitalizations due to LRTI or COVID-19 LRTI specifically are identified as risk factors for subsequent LRTI, patients could be targeted for immunization before discharge as part of routine hospital policy. Thus, we conducted an observational cohort study at Kaiser Permanente Southern California (KPSC) to examine the marginal increase in risk of new-onset LRTI after discharge among adults hospitalized for LRTI – including that due to COVID-19 – versus those hospitalized for any other cause.

## Methods

### Study population

KPSC is a large integrated health care system with 15 hospitals and associated outpatient facilities located across 7 Southern California counties. KPSC serves over 4.7 million members with diverse racial/ethnic and socioeconomic backgrounds similar to the underlying population [22]. Comprehensive electronic health records (EHR) capture all details of inpatient and outpatient care, including diagnoses, laboratory tests, and pharmacy prescription and dispensing records. Care received outside of KPSC is captured by insurance claims and integrated into EHR, facilitating longitudinal follow-up of members. The study was approved by the KPSC Institutional Review Board, which granted a waiver of informed consent and determined that the study posed minimal risk to study participants (IRB approval number 12496).

The study population included adults with a hospital discharge (index hospitalization) from 3/1/2020 to 5/31/2022, excluding individuals hospitalized with COVID-19 without LRTI and those admitted for labor and delivery. The index date (beginning of follow-up) was defined as the first day after a period of 30 days after hospital discharge without antibiotics to reduce the likelihood of capturing carry-over codes or codes associated with ongoing infection. Individuals were included in the study if they were aged ≥ 18 years at index date and had been a KPSC member for ≥ 1 year prior to the index hospitalization, which facilitated near-complete capture of demographic, care-seeking, and clinical characteristics. To allow the opportunity for adequate follow-up time, individuals were excluded if there was less than < 2 months of calendar-time after index date before the study end (8/31/2022).

### Exposure and outcome

We categorized individuals into 3 mutually exclusive baseline exposure groups: those hospitalized for COVID-19 LRTI, those hospitalized for non-COVID-19 LRTI, and those hospitalized for all other causes without COVID-19 or LRTI (“non-LRTI”) during the index hospitalization. We defined hospitalization for COVID-19 LRTI as hospitalization occurring within 7 days of a SARS-CoV-2 positive molecular test or hospitalization with a clinically confirmed COVID-19 diagnosis, along with hospitalization for LRTI, which was defined by discharge diagnoses using *International Classification of Diseases Tenth Revision (ICD-10)* codes J9-J18 (except J12.82 and J18.2) and J20-J22. Additionally, hospitalized patients with a discharge diagnosis using ICD-10 code J12.82 (pneumonia due to COVID-19) were categorized into the COVID-19 LRTI baseline exposure group without requiring a separate LRTI diagnosis code. We defined hospitalization for non-COVID-19 LRTI as hospitalization occurring with LRTI discharge diagnoses but without a SARS-CoV-2 positive molecular test within 7 days of hospitalization or a clinically confirmed COVID-19 diagnosis (including J12.82).

The outcome of interest was new-onset LRTI during follow-up, identified by ICD-10 codes J9-J18 (except J12.82 and J18.2) and J20-J22 in any care setting (outpatient, emergency departments [ED], and inpatient). Individuals were followed-up from index date until outcome of interest for up to 1 year, with censoring for occurrence of a positive SARS-CoV-2 test or COVID-19 diagnosis, disenrollment from KPSC, death, or end of study period (8/31/2022). If a positive SARS-CoV-2 test or COVID-19 diagnosis was recorded ≤ 90 days after discharge from the original COVID-19 LRTI hospitalization, this was considered related to the original index hospitalization and the patient was not censored.

### Other variables

We captured demographic and clinical characteristics from individuals’ EHR. These included: age, sex, race/ethnicity, prior year health care utilization (number of outpatient visits, number of emergency department [ED] visits, number of hospitalizations), prior year Elixhauser comorbidities, prior receipt of 13-valent pneumococcal conjugate vaccine (PCV13) or 23-valent pneumococcal polysaccharide vaccine (PPSV23), prior year receipt of influenza vaccine, prior receipt of COVID-19 vaccine, history of LRTI in the prior year, frailty (using the Hospital Frailty Risk Score described by Gilbert et al. [23]), and pneumococcal disease risk categories (high risk, medium risk, and low risk) as specified by ACIP as criteria for pneumococcal vaccination (S Table 1) [6, 24]. We further indicated whether admission to intensive care unit (ICU) and invasive mechanical ventilation occurred at the index hospitalization, and the month and year of admission. We also captured pneumococcal, influenza, and COVID-19 vaccinations during follow-up.

### Statistical analyses

We described patient characteristics among individuals hospitalized for COVID-19 LRTI, non-COVID-19 LRTI, or non-LRTI causes. We calculated the number of new-onset LRTI events during follow-up, person-time at risk, and rates of new-onset LRTI for each hospitalized group. Cumulative incidence plots of new-onset LRTI by hospitalized group were generated using nonparametric Aalen–Johansen estimator to account for mortality as a competing risk.

We used multivariable cause-specific Cox regression with time-varying covariates to estimate hazard ratios (HR) and 95% confidence intervals (CI) for new-onset LRTI diagnoses during follow-up separately for individuals hospitalized for COVID-19 LRTI and individuals hospitalized for non-COVID-19 LRTI compared to those hospitalized for non-LRTI causes, considering death as a competing risk. Multivariable models were adjusted for variables described above, with pneumococcal, influenza, and COVID-19 vaccination variables included as time-varying covariates. We also performed analyses stratified by key LRTI risk factors, including age group, ACIP-designated pneumococcal disease risk categories, and ICU status during index hospitalization. Analyses were performed using R statistical software version 4.0.4.

## Results

The study included 22,417 individuals hospitalized for COVID-19 LRTI, 12,795 individuals hospitalized for non-COVID-19 LRTI, and 176,788 individuals hospitalized for non-LRTI (Table 1). Individuals hospitalized for non-COVID-19 LRTI were older than those hospitalized for non-LRTI and those hospitalized for COVID-19 LRTI (median [interquartile range] age in years of 72 [61,81], 63 [45,75], and 60 [49,71] respectively). Compared to individuals hospitalized for non-LRTI, those hospitalized for COVID-19 LRTI were more commonly male, Hispanic and had fewer prior year healthcare encounters. Individuals hospitalized for non-COVID-19 LRTI were more commonly male, non-Hispanic White, had more prior year ED and inpatient visits, and had more comorbidities than those hospitalized for non-LRTI; they also more commonly had history of LRTI, higher frailty, and belonged to the ACIP high risk category. Admission to the ICU or receipt of invasive mechanical ventilation during the index hospitalization occurred more commonly among those hospitalized for COVID-19 LRTI and those hospitalized for non-COVID-19 LRTI than those hospitalized for non-LRTI causes.

**Table 1:** Baseline characteristics of individuals hospitalized at Kaiser Permanente Southern California from 3/1/2020 to 5/31/2022^a^

Characteristic	non-LRTI hospitalization *N* = 176,788 n (%)	COVID-19 LRTI hospitalization, *N* = 22,417 n (%)	Non-COVID-19 LRTI hospitalization, *N* = 12,795 n (%)
Age			
18–< 35 years	28,774 (16.3)	1,764 (7.9)	694 (5.4)
36–< 50 years	23,492 (13.3)	4,156 (18.5)	904 (7.1)
50–< 65 years	40,124 (22.7)	7,732 (34.5)	2,459 (19.2)
65–< 75 years	37,208 (21.0)	4,577 (20.4)	3,113 (24.3)
≥ 75 years	47,190 (26.7)	4,188 (18.7)	5,625 (44.0)
Sex			
F	95,422 (54.0)	9,531 (42.5)	5,888 (46.0)
M	81,366 (46.0)	12,886 (57.5)	6,907 (54.0)
Race/Ethnicity			
White	76,398 (43.8)	5,740 (26.1)	6,259 (49.5)
Asian	14,514 (8.3)	1,976 (9.0)	1,064 (8.4)
Black	19,408 (11.1)	2,076 (9.4)	1,515 (12.0)
Hispanic	61,063 (35.0)	11,840 (53.9)	3,666 (29.0)
Other/Unknown	2,871 (1.6)	339 (1.5)	137 (1.1)
Unknown	2,534	446	154
Number of outpatient encounters			
0–5	36,869 (20.9)	8,430 (37.6)	2,901 (22.7)
6–10	28,715 (16.2)	5,210 (23.2)	2,233 (17.5)
≥ 11	111,204 (62.9)	8,777 (39.2)	7,661 (59.9)
Number of ED encounters^b^			
0	99,304 (56.2)	13,541 (60.4)	6,505 (50.8)
1	42,636 (24.1)	5,820 (26.0)	3,197 (25.0)
≥ 2	34,848 (19.7)	3,056 (13.6)	3,093 (24.2)
Number of inpatient encounters^b^			
0	161,562 (91.4)	21,659 (96.6)	11,037 (86.3)
1	10,729 (6.1)	619 (2.8)	1,130 (8.8)
≥ 2	4,497 (2.5)	139 (0.6)	628 (4.9)
Elixhauser^b^			
0–2	85,622 (48.4)	13,674 (61.0)	3,962 (31.0)
3–5	55,755 (31.5)	6,075 (27.1)	4,240 (33.1)
6–8	25,762 (14.6)	2,076 (9.3)	3,056 (23.9)
≥ 9	9,649 (5.5)	592 (2.6)	1,537 (12.0)
Previous LRTI	10,877 (6.2)	3,830 (17.1)	2,661 (20.8)
Frailty index category			
< 5	103,644 (58.6)	16,348 (72.9)	5,798 (45.3)
5–< 15	56,350 (31.9)	5,003 (22.3)	4,700 (36.7)
≥ 15	16,794 (9.5)	1,066 (4.8)	2,297 (18.0)
ACIP risk category			
Low	77,424 (43.8)	10,557 (47.1)	3,385 (26.5)
Medium	65,056 (36.8)	8,242 (36.8)	5,325 (41.6)
High	34,308 (19.4)	3,618 (16.1)	4,085 (31.9)
ICU during index hospitalization	14,028 (7.9)	2,314 (10.3)	1,319 (10.3)
Ventilation during index hospitalization	3,470 (2.0)	1,077 (4.8)	633 (4.9)
PPSV23 receipt			
Before index-date	113,591 (64.3)	13,327 (59.5)	10,630 (83.1)
During follow-up	3,161 (1.8)	620 (2.8)	178 (1.4)
PCV13 receipt			
Before index-date	83,521 (47.2)	8,453 (37.7)	8,837 (69.1)
During follow-up	2,100 (1.2)	243 (1.1)	118 (0.9)
Flu vaccine			
In season prior to index date	100,043 (56.6)	11,310 (50.5)	7,928 (62.0)
During follow-up	28,172 (15.9)	3,192 (14.2)	1,778 (13.9)
COVID-19 vaccine			
1st dose prior to index date	9,124 (5.2)	1,302 (5.8)	688 (5.4)
1st dose during follow-up	50,001 (28.3)	11,560 (51.6)	3,052 (23.9)
1^st^ and 2^nd^ dose prior to index date	47,578 (26.9)	1,491 (6.7)	3,133 (24.5)
2^nd^ dose during follow-up	50,246 (28.4)	11,505 (51.3)	3,037 (23.7)
1^st^, 2^nd^, and 3^rd^ dose prior to index date	25,916 (14.7)	476 (2.1)	1,880 (14.7)
3^rd^ dose during follow-up	40,033 (22.6)	5,448 (24.3)	2,210 (17.3)

*Abbreviations*: *ACIP* Advisory Committee on Immunization Practices, *COVID-19* Coronavirus disease 2019, *ED* Emergency department, *ICU* Intensive care unit, *LRTI* Lower respiratory tract infection, *PCV13* 13-valent pneumococcal conjugate vaccine, *PPSV23* 23-valent pneumococcal polysaccharide vaccine

^a^Index hospitalizations include individuals hospitalized for reasons other than LRTI (“non-LRTI”), those hospitalized for COVID-19 LRTI (“COVID-19 LRTI”), and those hospitalized for LRTI without COVID-19 (“non-COVID-19 LRTI”)

^b^Defined in year prior to index hospital admission date

A greater proportion of individuals hospitalized for non-COVID-19 LRTI had received PPSV23, PCV13, and seasonal influenza vaccination prior to the index date (83.1%, 69.1%, and 62.0%, respectively) than those hospitalized for non-LRTI (64.3%, 47.2%, and 56.6%) and those hospitalized for COVID-19 LRTI (59.5%, 37.7%, 50.5%) (Table 1); however, during follow-up, < 3% of individuals in all groups received pneumococcal vaccines, and receipt of influenza vaccine was highest among those hospitalized for non-LRTI (15.9% vs 14.2% among those hospitalized for COVID-19 LRTI and 13.9% among those hospitalized for non-COVID-19 LRTI). Comparing those hospitalized for COVID-19 LRTI to those hospitalized for non-COVID-19 LRTI or non-LRTI, fewer had received COVID-19 vaccine 2 or 3 doses prior to the index date, but more received doses during follow-up.

**Fig. 1:**
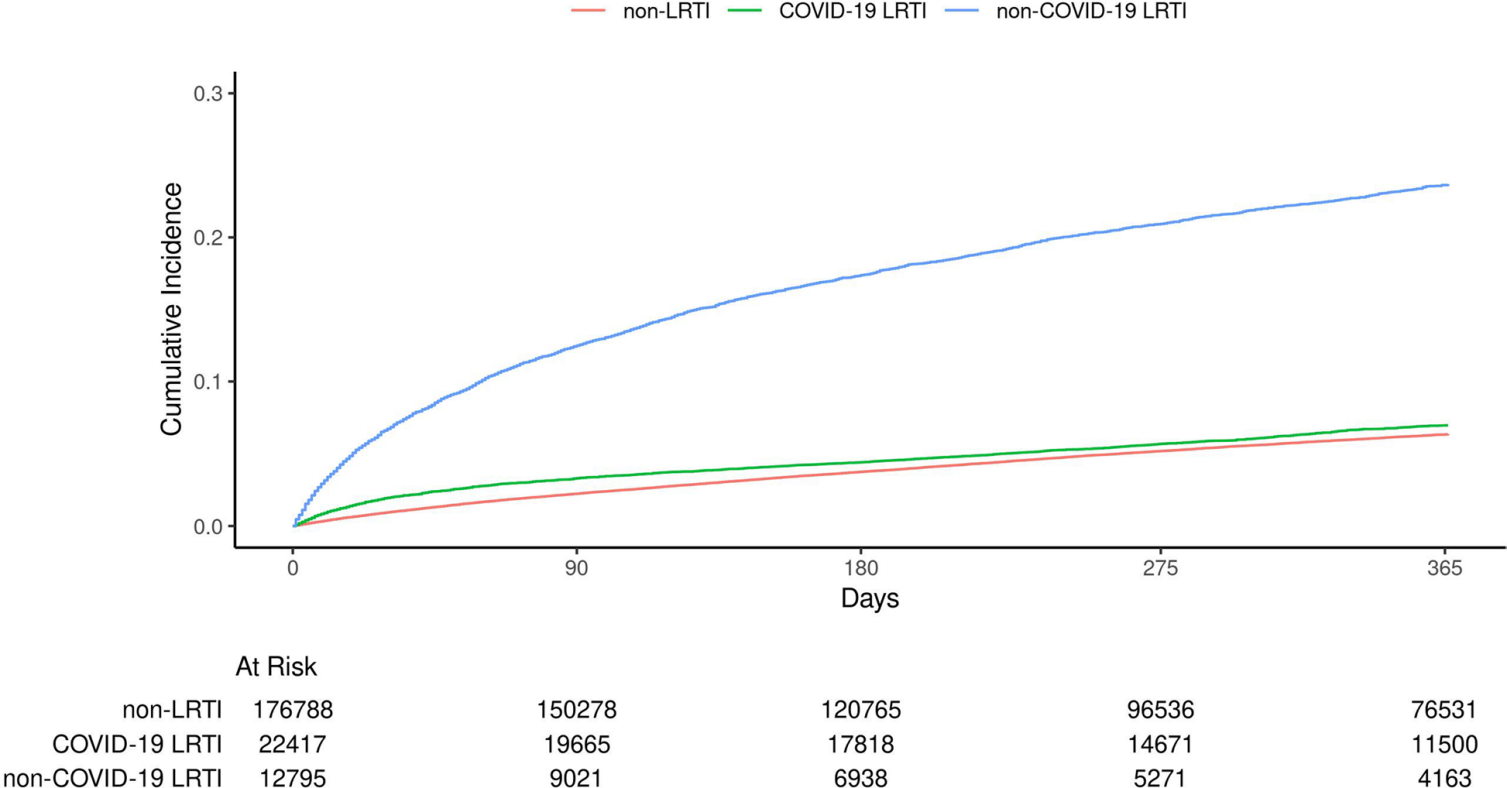
Cumulative incidence of new-onset LRTI after index hospitalization^a^. Abbreviations: COVID-19, coronavirus disease 2019; LRTI, lower respiratory tract infection. ^a^Index hospitalizations include individuals hospitalized for reasons other than LRTI (“non-LRTI”), those hospitalized for COVID-19 LRTI (“COVID-19 LRTI”), and those hospitalized for LRTI without COVID-19 (“non-COVID-19 LRTI”)

During follow-up, there were 1,348 new-onset LRTI events among individuals hospitalized for COVID-19 LRTI, 2,516 events among individuals hospitalized for non-COVID-19 LRTI, and 8,878 events among individuals hospitalized for non-LRTI, yielding unadjusted incidence rates (95% confidence intervals) per 1,000 person-years of 55.4 (52.5–58.4), 253.5 (243.7–263.6), and 52.5 (51.4,53.6), respectively (Table 2). Incidence rates of new-onset LRTI during follow-up among all 3 hospitalized groups were higher for individuals aged ≥ 65 years vs < 65 years and for those in the high-risk and medium-risk ACIP categories vs. the low-risk category. Incidence rates were slightly higher for those admitted to the ICU vs. not admitted to the ICU among individuals hospitalized for non-LRTI and those hospitalized for COVID-19 LRTI, but not for those hospitalized for non-COVID-19 LRTI. The unadjusted cumulative incidence of new-onset LRTI was highest for those hospitalized for non-COVID-19 LRTI (365-day cumulative incidence: 24% [95% CI: 23–24%]) and remained marginally higher for those hospitalized for COVID-19 LRTI (7.0% [6.6–7.4%]) compared to those hospitalized for non-LRTI (6.4% [6.2%, 6.5%]) (Fig. 1).

**Table 2:** Incidence rates of new-onset LRTI after index hospitalization by reason for hospitalization^a^

	**N**	**Events**	**Person-Years**	**Incidence per 1000 person-years (95% CI)**	**365-day Cumulative Incidence (95% CI)**^b^
**Full Cohort**					
Non-LRTI	176,788	8878	169,245.910	52.5 (51.4,53.6)	6.4% (6.2%, 6.5%)
COVID-19 LRTI	22,417	1348	24,345.871	55.4 (52.5,58.4)	7.0% (6.6%, 7.4%)
Non-COVID-19 LRTI	12,795	2516	9926.175	253.5 (243.7,263.6)	24% (23%, 24%)
**Age ≥ 65 years**					
Non-LRTI	84,398	6078	79,600.660	76.4 (74.4,78.3)	8.9% (8.6%, 9.1%)
COVID-19 LRTI	8765	761	9110.926	83.5 (77.7,89.7)	10% (9.5%, 11%)
Non-COVID-19 LRTI	8738	1802	6487.975	277.7 (265.1,290.9)	25% (24%, 26%)
**Age < 65 years**					
Non-LRTI	92,390	2800	89,645.25	31.2 (30.1,32.4)	3.9% (3.8%, 4.1%)
COVID-19 LRTI	13,652	587	15,234.95	38.5 (35.5,41.8)	5.0% (4.6%, 5.4%)
Non-COVID-19 LRTI	4057	714	3438.20	207.7 (192.7,223.5)	21% (19%, 22%)
**ICU during index hospitalization**					
Non-LRTI	14,028	814	13,828.277	58.9 (54.9,63.1)	7.2% (6.7%, 7.7%)
COVID-19 LRTI	2314	144	2445.827	58.9 (49.7,69.3)	7.4% (6.3%, 8.6%)
Non-COVID-19 LRTI	1319	253	1112.603	227.4 (200.2,257.2)	23% (20%, 26%)
**No ICU during index hospitalization**					
Non-LRTI	162,760	8064	155,417.633	51.9 (50.8,53)	6.3% (6.1%, 6.4%)
COVID-19 LRTI	20,103	1204	21,900.044	55 (51.9,58.2)	6.9% (6.6%, 7.3%)
Non-COVID-19 LRTI	11,476	2263	8813.573	256.8 (246.3,267.6)	24% (23%, 25%)
**ACIP high risk**					
Non-LRTI	34,308	3419	30,568.468	111.8 (108.1,115.7)	12% (12%, 13%)
COVID-19 LRTI	3618	407	3549.384	114.7 (103.8,126.4)	14% (12%, 15%)
Non-COVID-19 LRTI	4085	949	2954.408	321.2 (301.1,342.3)	28% (26%, 29%)
**ACIP medium risk**					
Non-LRTI	65,056	3699	62,371.378	59.3 (57.4,61.2)	7.2% (7.0%, 7.4%)
COVID-19 LRTI	8242	536	9093.688	58.9 (54.1,64.1)	7.5% (6.9%, 8.2%)
Non-COVID-19 LRTI	5325	1093	4147.288	263.5 (248.2,279.6)	25% (24%, 26%)
**ACIP low risk**					
Non-LRTI	77,424	1760	76,306.063	23.1 (22,24.2)	2.9% (2.8%, 3.1%)
COVID-19 LRTI	10,557	405	11,702.800	34.6 (31.3,38.1)	4.4% (4.0%, 4.8%)
Non-COVID-19 LRTI	3385	474	2824.479	167.8 (153,183.6)	16% (15%, 18%)

*Abbreviations*: *ACIP* Advisory Committee on Immunization Practices, *COVID-19* Coronavirus disease 2019, *ICU* Intensive care unit, *LRTI* Lower respiratory tract infection

^a^Index hospitalizations include individuals hospitalized for reasons other than LRTI (“non-LRTI”), those hospitalized for COVID-19 LRTI (“COVID-19 LRTI”), and those hospitalized for LRTI without COVID-19 (“non-COVID-19 LRTI”)

^b^365-day cumulative incidence of new-onset LRTI by hospitalized group estimated using nonparametric Aalen–Johansen estimator to account for mortality as a competing risk. Cumulative incidence estimates are estimated in terms of percentage of the study population, rather than person-years, and additionally account for mortality as a competing risk, so they are not directly comparable with other incidence estimates in this table

**Fig. 2:**
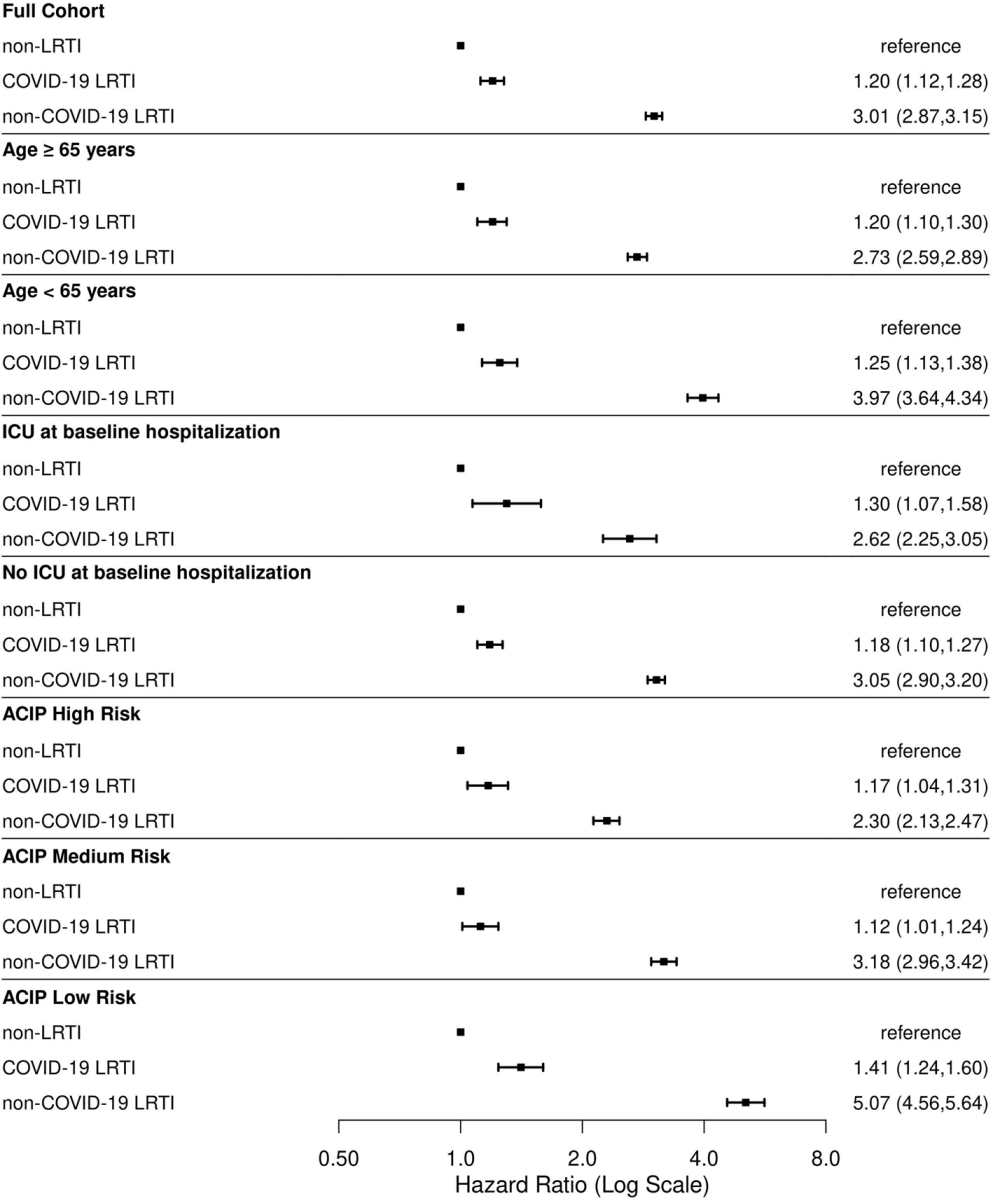
Hazard ratios for new-onset LRTI after index hospitalization^a^. Abbreviations: ACIP, Advisory Committee on Immunization Practices; COVID-19, coronavirus disease 2019; ICU, intensive care unit; LRTI, lower respiratory tract infection. ^a^Index hospitalizations include individuals hospitalized for reasons other than LRTI (“non-LRTI”), those hospitalized for COVID-19 LRTI (“COVID-19 LRTI”), and those hospitalized for LRTI without COVID-19 (“non-COVID-19 LRTI”)

In adjusted analyses, individuals hospitalized for COVID-19 LRTI had a 20% higher hazard of new-onset LRTI during follow-up compared to those hospitalized for non-LRTI (HR [95% CI] 1.20 [1.12–1.28] (Fig. 2). Individuals hospitalized for non-COVID-19 LRTI had a 301% higher hazard of new-onset LRTI during follow-up compared to those hospitalized for non-LRTI (HR 3.01 [2.87–3.15]). In stratified analyses, the relative hazard of new-onset LRTI comparing those hospitalized for LRTI vs. non-LRTI tended to be higher among individuals with low vs. high LRTI risk. For example, when comparing individuals hospitalized for non-COVID-19 LRTI vs. non-LRTI, the relative hazard of new-onset LRTI was higher for individuals aged < 65 years (HR 3.97 [3.64–4.34]) than for those aged ≥ 65 years (HR 2.62 [2.25–3.05]) and for those in the low-risk ACIP stratum for pneumococcal disease (HR 5.07 [4.56–5.64]) than for those in the high risk stratum (HR 2.30 [2.13–2.47]).

## Discussion

In this large cohort study, all hospitalized individuals had a high risk of new-onset LRTI during follow-up. A separate study from the KPSC population prior to the COVID-19 pandemic reported that the incidence of LRTI among individuals aged ≥ 65 years was 45 to 53 per 1000 person-years of follow-up (depending on PCV13 vaccination status) [25]. In our study, individuals aged ≥ 65 years hospitalized for non-LRTI had a new-onset LRTI incidence of 76 per 1000 person-years of follow-up, approximately 1.5 times the overall background LRTI risk in this population. Individuals aged ≥ 65 years hospitalized for COVID-19 LRTI and those hospitalized for non-COVID-19 LRTI had incidences of 84 and 278 per 1000 person-years of follow-up, or approximately 1.5- to 5-fold higher than background risk. Aligning with other evidence [26], our data indicate that hospitalization itself, and especially LRTI due to any cause, is a marker for risk of a new-onset LRTI and that hospitalized individuals might be targeted for vaccines active against respiratory infections such as influenza, RSV, and pneumococcal conjugate vaccines.

In our study, when compared to individuals hospitalized for non-LRTI, individuals hospitalized for non-COVID-19 LRTI had a higher risk than those hospitalized for COVID-19 LRTI of having subsequent LRTI. Two broad hypotheses exist to explain this finding. First, SARS-CoV-2 may cause less damage to lungs than other etiologies of LRTI such as influenza, RSV, or pneumococci. However, a large body of evidence supports damage from SARS-CoV-2 to lung parenchyma, undermining the plausibility of this explanation [10–19]. Second, individuals hospitalized for COVID-19 LRTI may represent a population with substantially lower a priori risk for pneumonia than individuals hospitalized for non-COVID-19 LRTI. This hypothesis is strongly supported by our study. Compared to people hospitalized for COVID-19 LRTI, those with non-COVID-19 LRTI were older, had more comorbidities (e.g., they were approximately 1.5-fold more likely to have ≥ 6 Elixhauser comorbidities), were frailer (e.g., 4-fold more likely to be in the most frail category), and had more hospitalizations in the prior year (e.g., 8-fold more likely to have had ≥ 2 prior hospitalizations). Although we adjusted for these factors in multivariable analysis, it is likely that we could not fully account for residual confounding. In sum, rather than illustrating that SARS-CoV-2 causes less lung damage than other viruses, our data suggest that SARS-CoV-2 leading to hospitalization can increase risk of LRTI even among persons with substantially lower a priori risk of this outcome.

The US CDC and expert groups in many other countries define persons at risk for pneumococcal disease based on underlying conditions such as immunodeficiency, chronic lung disease, congestive heart failure, and others [6, 24]. In our study, hospitalized individuals in all three study groups who were designated as being at medium risk or at high risk for invasive pneumococcal disease had substantially higher incidences of subsequent new-onset LRTI compared to those in the ACIP low risk stratum (immunocompetent individuals without major medical comorbidities). However, the relative increase in risk of new-onset LRTI for individuals hospitalized for COVID-19 or non-COVID-19 LRTI vs. non-LRTI was highest in low-risk groups. This finding may have resulted from the low risk of subsequent hospitalization among low-risk adults hospitalized for non-LRTI causes. Regardless of the cause, these findings suggest that individuals hospitalized for LRTI, even those who are otherwise at low risk of invasive pneumococcal disease, may benefit from receiving vaccines targeting respiratory pathogens. More generally, a strategy of ensuring that adults hospitalized for any reason – and particularly for pneumonia/LRTI – have access to immunization against respiratory diseases prior to hospital discharge may be warranted.

Our study has several limitations. First, our study was focused on a hospitalized cohort, and thus we could not assess the risk of subsequent LRTI among our three study groups compared directly to LRTI risk among subjects who were not hospitalized. This limitation is partially mitigated by a recent publication on background pneumonia/LRTI risk among the underlying population at KPSC prior to the COVID-19 pandemic [25]. Second, despite reduced circulation of many respiratory pathogens during the follow-up period, rates of new-onset LRTI in our study were higher in comparison to rates of subsequent community acquired pneumonia (CAP) reported in limited other studies [27–29]. This may be because we included a broader range of LRTI diagnoses, but it is also possible that individuals in our study sought more frequent care for respiratory symptoms after hospitalization, spurred by concerns about COVID-19. Alternatively, it is possible that some of the diagnoses of LRTI during follow-up were related to the initial infection and not a new infection. To limit recrudescent or nosocomial infections, we did not begin follow-up until 30 days after hospital discharge without antibiotics in supply. Diagnosis codes may have been carried forward, but this is less likely for acute pneumonia/LRTI than for chronic conditions. Third, although we adjusted for multiple demographic and clinical covariates at baseline, we could not capture all clinical characteristics or behavioral or community factors that might have differed by hospitalization group and that also influenced risk of LRTI during follow-up. Fifth, we were unable to determine the etiology of index or subsequent LRTI events, as these data are not consistently captured in EHR.

## Conclusion

Results of this study in a large, diverse cohort of hospitalized patients found that adults hospitalized for any reason have a high risk of subsequent new-onset LRTI and that this risk is accentuated among individuals hospitalized for non-COVID-19 LRTI. Moreover, our study found that individuals hospitalized for COVID-19 LRTI had a high risk of subsequent new-onset LRTI despite representing a relatively healthy population when compared to the older, more frail population hospitalized with non-COVID-19 LRTI. While our study design precluded identification of specific etiologies, our findings support consideration of hospitalization for any reason – and particularly hospitalization for LRTI – as an indication for immunization with vaccines active against respiratory pathogens.

### Supplementary Information

**Additional file 1: S Table 1.** Risk categories for pneumococcal vaccination based on US Advisory Committee on Immunization Practices criteria.

## Data Availability

Anonymized data that support the findings of this study may be made available from the investigative team in the following conditions: (1) agreement to collaborate with the study team on all publications, (2) provision of external funding for administrative and investigator time necessary for this collaboration, (3) demonstration that the external investigative team is qualified and has documented evidence of training for human subjects protections, and (4) agreement to abide by the terms outlined in data use agreements between institutions.

## Abbreviations

ACIP: Advisory Committee on Immunization Practices
CDC: Centers for Disease Control and Prevention
COVID-19: Coronavirus disease 2019
ED: Emergency department
HER: Electronic health record
ICD-10: International Classification of Diseases Tenth Revision
ICU: Intensive care unit
KPSC: Kaiser Permanente Southern California
LRTI: Lower respiratory tract infection
PCV: Pneumococcal conjugate vaccine
PPSV23: 23-Valent pneumococcal polysaccharide vaccine
SARS-CoV-2: Severe acute respiratory syndrome coronavirus 2
