# Supplementary material for: Risk of subsequent lower respiratory tract infection (LRTI) after hospitalization for COVID-19 LRTI and non-COVID-19 LRTI: a retrospective cohort study
Source: Pneumonia (Nathan). 2023 Oct 5;15:15. doi: 10.1186/s41479-023-00117-5 (PMC10552217; doi:10.1186/s41479-023-00117-5)
Supplement: Supplementary file 1 — Additional file 1: S Table 1. Risk categories for pneumococcal vaccination based on US Advisory Committee on Immunization Practices criteria. [file 41479_2023_117_MOESM1_ESM.docx]

**Supplementary information for:**

**Risk of subsequent lower respiratory tract infection (LRTI) after hospitalization for COVID-19 LRTI and non-COVID-19 LRTI: a retrospective cohort study**

**Authors:** Katia J. Bruxvoort^1, 2*^, Heidi Fischer^1^, Joseph A. Lewnard^3, 4, 5^, Vennis X. Hong^1^, Magdalena Pomichowski^1^, Lindsay R. Grant^6^, Luis Jódar^6^, Bradford D. Gessner^6^, Sara Y. Tartof^1^

**Affiliations:**

1. Department of Research & Evaluation, Kaiser Permanente Southern California, Pasadena, California, United States
2. Department of Epidemiology, School of Public Health, University of Alabama at Birmingham, Birmingham, Alabama, United States
3. Division of Epidemiology, School of Public Health, University of C0alifornia, Berkeley, Berkeley, California, United States
4. Division of Infectious Diseases & Vaccinology, School of Public Health, University of California, Berkeley, Berkeley, California, United States
5. Center for Computational Biology, College of Engineering, University of California, Berkeley, Berkeley, California, United States
6. Pfizer Vaccines, Collegeville, Pennsylvania, United States

*Correspondence to: Katia Bruxvoort, 1665 University Blvd, Birmingham, Alabama, 35233, +1 (205) 975-8626; [kbruxvoort@uab.edu](mailto:kbruxvoort@uab.edu)

**S Table 1: Risk categories for pneumococcal vaccination based on US Advisory Committee on Immunization Practices criteria^1^**

| **Condition** | **ICD-10 Codes** |
| --- | --- |
| **High risk** |  |
| Cerebrospinal fluid leak | G97.0, G96.0 |
| Cochlear implant | Z96.21, Z96.20 |
| Functional or anatomic asplenia | Q89.01, Z90.81 |
| Sickle cell disease / other hemaglobinopathy | D57, D57.0, D57.00, D57.01, D57.02, D57.1, D57.2, D57.20, D57.21, D57.211, D57.212, D57.219 |
| Congenital or acquired immunodeficiency | D80, D80.0, D80.1, D80.2, D80.3, D80.4, D80.5, D80.6, D80.7, D80.8, D80.9, D81, D81.0, D81.1, D81.2, D81.3, D81.4, D81.5, D81.6, D81.7, D81.8, D81.81, D81.810, D81.818, D81.819, D81.89, D81.9, D82, D82.0, D82.1, D82.2, D82.3, D82.4, D82.8, D82.9, D83, D83.0, D83.1, D83.2, D83.1, D83.2, D83.8, D83.9, D84, D84.0, D84.1, D84.8, D84.9 |
| HIV infection | B20, Z21 |
| Chronic renal failure | D63.1, E08.2, E08.21, E08.22, E08.29, E09.2, E09.21, E09.22, E09.29, E10.2, E10.21, E10.22, E10.29, E11.2, E11.21, E11.22, E11.29, E13.2, E13.21, E13.22, E13.29, E879.1, I12.0, I13.11, I13.2, I95.3, M32.14, M32.15, M35.04, N03, N03.0, N03.1, N03.2, N03.3, N03.4, N03.5, N03.6, N03.7, N03.8, N03.9, N05, N05.0, N05.1, N05.2, N05.3, N05.4, N05.5, N05.6, N05.7, N05.8, N05.9, N06, N06.0, N06.1, N06.2, N06.3, N06.4, N06.5, N06.6, N06.7, N06.8, N06.9, N07, N07.0, N07.1, N07.2, N07.3, N07.4, N07.5, N07.6, N07.7, N07.8, N07.9, N08, N13.72, N13.721, N13.722, N13.729, N13.73, N13.731, N13.732, N13.739, N13.9, N14, N14.0, N14.1, N14.2, N14.3, N14.4, N15.0, N15.8, 15.9, N16, N18.5, N18.6, N19, N25.0, N25.81, N25.89, N25.9, R88.0, T85.611, T85.611A, T85.611D, T85.611S, T85.621, T85.621A, T85.621D, T85.621S, T85.631, T85.631A, T85.631D, T85.631S, T85.691, T85.691A, T85.691D, T85.691S, Y84.1, Z49, Z49.0, Z49.01, Z49.02, Z49.3, Z49.31, Z49.32, Z91.15, Z99.2 |
| Nephrotic syndrome | N04.0, N04.1, N04.2, N04.3, N04.4, N04.5, N04.6, N04.7, N04.8, N04.9, Z87.441 |
| Leukemia, Lymphoma, Hodgkin disease, Generalized malignancy, Multiple myeloma | C80.0, C81.00, C81.01, C81.02, C81.03, C81.04, C81.05, C81.06, C81.07, C81.08, C81.09, C81.10, C81.11, C81.12, C81.13, C81.14, C81.15, C81.16, C81.17, C81.18, C81.19, C81.20, C81.21, C81.22, C81.23, C81.24, C81.25, C81.26, C81.27, C81.28, C81.29, C81.30, C81.31, C81.32, C81.33, C81.34, C81.35, C81.36, C81.37, C81.38, C81.39, C81.40, C81.41, C81.42, C81.43, C81.44, C81.45, C81.46, C81.47, C81.48, C81.49, C81.70, C81.71, C81.72, C81.73, C81.74, C81.75, C81.76, C81.77, C81.78, C81.79, C81.90, C81.91, C81.92, C81.93, C81.94, C81.95, C81.96, C81.97, C81.98, C81.99, C82.00, C82.01, C82.02, C82.03, C82.04, C82.05, C82.06, C82.07, C82.08, C82.09, C82.10, C82.11, C82.12, C82.13, C82.14, C82.15, C82.16, C82.17, C82.18, C82.19, C82.20, C82.21, C82.22, C82.23, C82.24, C82.25, C82.26, C82.27, C82.28, C82.29, C82.30, C82.31, C82.32, C82.33, C82.34, C82.35, C82.36, C82.37, C82.38, C82.39, C82.40, C82.41, C82.42, C82.43, C82.44, C82.45, C82.46, C82.47, C82.48, C82.49, C82.50, C82.51, C82.52, C82.53, C82.54, C82.55, C82.56, C82.57, C82.58, C82.59, C82.60, C82.61, C82.62, C82.63, C82.64, C82.65, C82.66, C82.67, C82.68, C82.69, C82.80, C82.81, C82.82, C82.83, C82.84, C82.85, C82.86, C82.87, C82.88, C82.89, C82.90, C82.91, C82.92, C82.93, C82.94, C82.95, C82.96, C82.97, C82.98, C82.99, C83.00, C83.01, C83.02, C83.03, C83.04, C83.05, C83.06, C83.07, C83.08, C83.09, C83.10, C83.11, C83.12, C83.13, C83.14, C83.15, C83.16, C83.17, C83.18, C83.19, C83.30, C83.31, C83.32, C83.33, C83.34, C83.35, C83.36, C83.37, C83.38, C83.39, C83.50, C83.51, C83.52, C83.53, C83.54, C83.55, C83.56, C83.57, C83.58, C83.59, C83.70, C83.71, C83.72, C83.73, C83.74, C83.75, C83.76, C83.77, C83.78, C83.79, C83.80, C83.81, C83.82, C83.83, C83.84, C83.85, C83.86, C83.87, C83.88, C83.89, C83.90, C83.91, C83.92, C83.93, C83.94, C83.95, C83.96, C83.97, C83.98, C83.99, C84.00, C84.01, C84.02, C84.03, C84.04, C84.05, C84.06, C84.07, C84.08, C84.09, C84.10, C84.11, C84.12, C84.13, C84.14, C84.15, C84.16, C84.17, C84.18, C84.19, C84.40, C84.41, C84.42, C84.43, C84.44, C84.45, C84.46, C84.47, C84.48, C84.49, C84.60, C84.61, C84.62, C84.63, C84.64, C84.65, C84.66, C84.67, C84.68, C84.69, C84.70, C84.71, C84.72, C84.73, C84.74, C84.75, C84.76, C84.77, C84.78, C84.79, C84.90, C84.91, C84.92, C84.93, C84.94, C84.95, C84.96, C84.97, C84.98, C84.99, C84.A0, C84.A1, C84.A2, C84.A3, C84.A4, C84.A5, C84.A6, C84.A7, C84.A8, C84.A9, C84.Z0, C84.Z1, C84.Z2, C84.Z3, C84.Z4, C84.Z5, C84.Z6, C84.Z7, C84.Z8, C84.Z9, C85.10, C85.11, C85.12, C85.13, C85.14, C85.15, C85.16, C85.17, C85.18, C85.19, C85.20, C85.21, C85.22, C85.23, C85.24, C85.25, C85.26, C85.27, C85.28, C85.29, C85.80, C85.81, C85.82, C85.83, C85.84, C85.85, C85.86, C85.87, C85.88, C85.89, C85.90, C85.91, C85.92, C85.93, C85.94, C85.95, C85.96, C85.97, C85.98, C85.99, C86.0, C86.1, C86.2, C86.3, C86.4, C86.5, C86.6, C88.0, C88.2, C88.3, C88.4, C88.8, C88.9, C90.00, C90.01, C90.02, C90.10, C90.12, C90.20, C90.22, C90.30, C90.32, C91.00, C91.02, C91.10, C91.12, C91.30, C91.32, C91.40, C91.42, C91.50, C91.52, C91.60, C91.62, C91.90, C91.92, C91.A0, C91.A2, C91.Z0, C91.Z2, C92.00, C92.02, C92.10, C92.12, C92.20, C92.22, C92.30, C92.32, C92.40, C92.42, C92.50, C92.52, C92.60, C92.62, C92.90, C92.92, C92.A0, C92.A2, C92.Z0, C92.Z2, C93.00, C93.02, C93.10, C93.12, C93.30, C93.32, C93.90, C93.92, C93.Z0, C93.Z2, C94.00, C94.02, C94.20, C94.22, C94.30, C94.32, C94.40, C94.42, C94.6, C94.80, C94.82, C95.00, C95.02, C95.10, C95.12, C95.90, C95.92, C96.0, C96.2, C96.4, C96.5, C96.6, C96.9, C96.A, C96.Z, Z51.11 |
| Iatrogenic immunosuppression^1^ | Z79.52 |
| Solid organ transplant | Z94.1, Z94.0, Z94.4, Z94.3, Z94.2, Z94.83, Z94.82, Z94.89 |
| **Medium risk** |  |
| Diabetes | E08.00, E08.01, E08.10, E08.11, E08.21, E08.22, E08.29, E08.311, E08.319, E08.3211, E08.3212, E08.3213, E08.3219, E08.3291, E08.3292, E08.3293, E08.3299, E08.3311, E08.3312, E08.3313, E08.65, E09.10, E08.3319, E08.3391, E08.3392, E08.3393, E08.3399, E08.3411, E09.01, E08.3412, E08.3413, E08.3419, E08.3491, E08.3492, E08.3493, E09.11, E09.65, E08.3499, E08.3511, E08.3512, E08.3513, E08.3519, E08.3521, E08.3522, E08.3523, E08.3529, E09.21, E08.3531, E08.3532, E08.3533, E08.3539, E08.3541, E08.3542, E08.3543, E08.3549, E08.3551, E08.3552, E08.3553, E08.3559, E08.3591, E08.3592, E08.3593, E08.3599, E08.36, E08.37X1, E08.37X2, E08.37X3, E08.37X9, E08.39, E08.40, E08.41, E08.42, E08.43, E08.44, E08.49, E08.51, E08.52, E08.59, E08.610, E08.618, E08.620, E08.621, E08.622, E08.628, E08.630, E08.638, E08.641, E08.649, E08.69, E08.8, E08.9, E09.00, E09.22, E09.29, E09.311, E09.319, E09.3211, E09.3212, E09.3213, E09.3219, E09.3291, E09.3292, E09.3293, E09.3299, E09.3311, E09.3312, E09.3313, E09.3319, E09.3391, E09.3392, E09.3393, E09.3399, E09.3411, E09.3412, E09.3413, E09.3419, E09.3491, E09.3492, E09.3493, E09.3499, E09.3511, E09.3512, E09.3513, E09.3519, E09.3521, E09.3522, E09.3523, E09.3529, E09.3531, E09.3532, E09.3533, E09.3539, E09.3541, E09.3542, E09.3543, E09.3549, E09.3551, E09.3552, E09.3553, E09.3559, E09.3591, E09.3592, E09.3593, E09.3599, E09.36, E09.37X1, E09.37X2, E09.37X3, E09.37X9, E09.39, E09.40, E09.41, E09.42, E09.43, E09.44, E09.49, E09.51, E09.52, E09.59, E09.610, E09.618, E09.620, E09.621, E09.622, E09.628, E09.630, E09.638, E09.641, E09.649, E09.69, E09.8, E09.9, E10.10, E10.11, E10.21, E10.22, E10.29, E10.311, E10.319, E10.3211, E10.3212, E10.3213, E10.3219, E10.3291, E10.3292, E10.3293, E10.3299, E10.3311, E10.3312, E10.3313, E10.3319, E10.3391, E10.3392, E10.3393, E10.3399, E10.3411, E10.3412, E10.3413, E10.3419, E10.3491, E10.3492, E10.3493, E10.3499, E10.3511, E10.3512, E10.3513, E10.3519, E10.3521, E10.3522, E10.3523, E10.3529, E10.3531, E10.3532, E10.3533, E10.3539, E10.3541, E10.3542, E10.3543, E10.3549, E13.40, E10.3551, E13.42, E10.3552, E10.3553, E10.3559, E10.3591, E10.3592, E10.3593, E10.3599, E10.36, E10.37X1, E10.37X2, E10.37X3, E10.37X9, E10.39, E10.40, E10.41, E10.42, E10.43, E10.44, E10.49, E10.51, E10.52, E10.59, E10.610, E10.618, E10.620, E10.621, E10.622, E10.628, E10.630, E10.638, E10.641, E10.649, E10.65, E10.69, E10.8, E10.9, E11.00, E11.01, E11.10, E11.11, E11.21, E11.22, E11.29, E11.311, E11.319, E13.9, E11.3211, E11.3212, E11.3213, E11.3219, E11.3291, E13.10, E11.3292, E11.65, E11.69, E11.3293, E11.3299, E13.00, E13.01, E11.3311, E11.3312, E11.3313, E13.11, E13.641, E11.3319, E11.3391, E11.3392, E11.3393, E11.3399, E13.21, E13.22, E13.29, E11.3411, E11.3412, E11.3413, E11.3419, E11.3491, E11.3492, E11.3493, E11.3499, E11.3511, E11.3512, E11.3513, E11.3519, E11.3521, E11.3522, E11.3523, E11.3529, E11.3531, E11.3532, E11.3533, E11.3539, E11.3541, E11.3542, E11.3543, E11.3549, E11.3551, E11.3552, E11.3553, E11.3559, E11.3591, E11.3592, E11.3593, E11.3599, E11.36, E11.37X1, E11.37X2, E11.37X3, E11.37X9, E11.39, E11.40, E11.41, E11.42, E11.43, E11.44, E11.49, E11.51, E11.52, E11.59, E11.610, E11.618, E11.620, E11.621, E11.622, E11.628, E11.630, E11.638, E11.641, E11.649, E13.311, E13.319, E13.3211, E13.3212, E13.3213, E13.3219, E13.3291, E13.3292, E13.3293, E13.3299, E13.3311, E13.3312, E13.3313, E13.3319, E13.3391, E13.3392, E13.3393, E13.3399, E13.3411, E13.3412, E13.3413, E13.3419, E13.3491, E13.3492, E13.3493, E13.3499, E13.3511, E13.3512, E13.3513, E13.3519, E13.3521, E13.3522, E13.3523, E13.3529, E13.3531, E13.3532, E13.3533, E13.3539, E13.3541, E13.3542, E13.3543, E13.3549, E13.3551, E13.3552, E13.3553, E13.3559, E13.3591, E13.3592, E13.3593, E13.3599, E13.36, E13.37X1, E13.37X2, E13.37X3, E13.37X9, E13.39, E11.8, E11.9, E13.41, E13.43, E13.44, E13.49, E13.610, E13.51, E13.52, E13.59, E13.618, E13.620, E13.621, E13.622, E13.628, E13.630, E13.638, E13.649, E13.65, E13.69, E13.8 |
| Chronic heart disease | A18.84, A18.84, I09.2, I20.0, I20.1, I20.8, I20.9, I21.01, I21.02, I21.09, I21.11, I21.19, I21.21, I21.29, I21.3, I21.4, I21.9, I21.A1, I21.A9, I22.0, I22.1, I22.2, I22.8, I22.9, I23.0, I23.1, I23.2, I23.3, I23.6, I23.7, I23.8, I24.0, I24.1, I24.8, I24.9, I25.10, I25.110, I25.111, I25.118, I25.119, I25.3, I25.41, I25.42, I25.5, I25.6, I25.700, I25.701, I25.708, I25.709, I25.710, I25.711, I25.718, I25.719, I25.720, I25.721, I25.728, I25.729, I25.730, I25.731, I25.738, I25.739, I25.750, I25.751, I25.758, I25.759, I25.760, I25.761, I25.768, I25.769, I25.790, I25.791, I25.798, I25.799, I25.810, I25.811, I25.812, I25.82, I25.83, I25.84, I25.89, I25.9, I34.0, I34.1, I34.2, I34.8, I34.9, I35.0, I35.1, I35.2, I35.8, I35.9, I36.0, I36.1, I36.2, I36.8, I36.9, I37.0, I37.1, I37.2, I37.8, I37.9, I38, I39, I42.0, I42.1, I42.2, I42.3, I42.4, I42.5, I42.6, I42.7, I42.8, I42.9, I43, I50.1, I50.20, I50.21, I50.22, I50.23, I50.30, I50.31, I50.32, I50.33, I50.40, I50.41, I50.42, I50.43, I50.810, I50.811, I50.812, I50.813, I50.814, I50.82, I50.83, I50.84, I50.89, I50.9, I51.0, M32.11 |
| Chronic lung disease | I27.20, I27.21, I27.22, I27.23, I27.24, I27.29, I27.81, I27.89, I27.9, J40, J44.9, J60, J61, J62.0, J62.8, J63.0, J63.1, J63.2, J63.3, J63.4, J63.5, J63.6, J64, J65, J66.0, J66.1, J66.2, J66.8, J68.4, J84.10, J84.17, J84.89, J96.10, J96.11, J96.12, J96.20, J96.21, J96.22, P27.0, P27.1, P27.8, P27.9 |
| Chronic liver disease | I85.00, I85.01, K70.0, K70.10, K70.11, K70.2, K70.30, K70.31, K70.40, K70.41, K70.9, K71.11, K72.01, K72.10, K72.11, K72.90, K72.91, K73.0, K73.1, K73.2, K73.8, K73.9, K74.0, K74.1, K74.2, K74.3, K74.4, K74.5, K74.60, K74.69, K75.0, K75.1, K75.4, K75.81, K76.0, K76.1, K76.6, K76.7, K76.89, K76.9 |
| Alcoholism | F10.121, F10.14, F10.150, F10.151, F10.159, F10.180, F10.181, F10.182, F10.188, F10.19, F10.221, F10.230, F10.231, F10.232, F10.239, F10.24, F10.250, F10.251, F10.259, F10.26, F10.27, F10.280, F10.281, F10.282, F10.288, F10.29, F10.920, F10.921, F10.929, F10.94, F10.950, F10.951, F10.959, F10.96, F10.97, F10.980, F10.981, F10.982, F10.988, F10.99, Z65.8 |
| Smoking | F17.200, F17.201, F17.210, F17.211, F17.220, F17.221, F17.290, F17.291, Z87.891 |

^1^High and medium risk defined as presence of any of these codes. Individuals who had codes for both high and medium risk categories were considered at high risk. Low risk was defined as the absence of these codes.
